# Multiple Single-Unit Long-Term Tracking on Organotypic Hippocampal Slices Using High-Density Microelectrode Arrays

**DOI:** 10.3389/fnins.2016.00537

**Published:** 2016-11-22

**Authors:** Wei Gong, Jure Senčar, Douglas J. Bakkum, David Jäckel, Marie Engelene J. Obien, Milos Radivojevic, Andreas R. Hierlemann

**Affiliations:** ^1^Bio Engineering Laboratory, Department of Biosystems Science and EngineeringETH Zürich, Basel, Switzerland; ^2^Faculty of Electrical Engineering, University of LjubljanaLjubljana, Slovenia

**Keywords:** organotypic slice, hippocampus, HD-MEA recording, new device, neuron tracking

## Abstract

A novel system to cultivate and record from organotypic brain slices directly on high-density microelectrode arrays (HD-MEA) was developed. This system allows for continuous recording of electrical activity of specific individual neurons at high spatial resolution while monitoring at the same time, neuronal network activity. For the first time, the electrical activity patterns of single neurons and the corresponding neuronal network in an organotypic hippocampal slice culture were studied during several consecutive weeks at daily intervals. An unsupervised iterative spike-sorting algorithm, based on PCA and k-means clustering, was developed to assign the activities to the single units. Spike-triggered average extracellular waveforms of an action potential recorded across neighboring electrodes, termed “footprints” of single-units were generated and tracked over weeks. The developed system offers the potential to study chronic impacts of drugs or genetic modifications on individual neurons in slice preparations over extended times.

**Key Concepts**

**Electrical-activity map:** A heat map that is plotted according to the largest absolute values of the negative amplitudes of extracellular action potentials on each electrode.

**Footprints:** A single-unit footprint represents the spatiotemporal extracellular waveforms of a single neuron, which has been calculated by spike-triggered averaging of signals determined according to spike times at the electrode with the largest signal.

**Central footprint peak amplitude *(CF amplitude)*:** the absolute value of the largest negative spike amplitude, within the footprint.

**Central footprint location *(CF location)*:** the location of the largest amplitude-signal within the footprint.

**Central footprint area *(CF area)*:** the area of footprints that included electrodes with negative spike amplitudes larger than 50% of the *CF amplitude*.

## Introduction

An important direction in neuroengineering is the development of new techniques and methods to observe the activity of as many neurons as possible, ideally within intact networks at high spatial resolution, and within a large frequency range over long time periods (Marblestone et al., 2013). Such techniques would allow for analyzing how individual neurons interact, and how their activities contribute to the overall network activity. High spatial-resolution recordings enable the identification and localization of single neurons, while a broad recording frequency range enables capturing neural activities at different time scales. By developing novel tools and methods, it will be possible to observe the dynamics and details of the activities of multiple individual neurons within a network environment, over extended times of days or weeks. Organotypic brain slice cultures constitute as an *ex vivo* system, and bridge the gap between dissociated cell cultures and *in vivo* animal experiments. Organotypic hippocampal slice cultures partially preserve the cytoarchitecture, synaptic circuits, and chemical signaling of the hippocampus (Gähwiler, 1981; Stoppini et al., 1991; Bahr, 1995; Gähwiler et al., 1997; De Paola et al., 2003; De Simoni and Yu, 2006; Galimberti et al., 2006). Organotypic slice preparations offer a large time window (i.e., up to 6 months) to investigate the functions of and changes in neural circuits during developmental processes (Claiborne et al., 1986; Henze et al., 2000), or changes that have been induced by chronic pharmacological (Stoppini et al., 1997; Noraberg, 2004; Cho et al., 2007; Baraban et al., 2009) and genetic manipulations (Ridoux et al., 1995; Thomas et al., 1998; Murphy and Messer, 2001; Duff et al., 2002; Jansen et al., 2011). However, long-term observation also requires a noninvasive recording method. Due to the invasive nature of, for instance the patch-clamp method, the slice cultures typically can only be recorded from during a single experimental trial (Stoppini et al., 1991; De Simoni et al., 2003) of up to 48 h (Dong and Buonomano, 2005). Long-term optical imaging of organotypic slice cultures is possible, and has been used to investigate synaptic morphology (Gogolla et al., 2006; Seidl and Rubel, 2010), however, the relatively low temporal resolution of fluorescence imaging methods may become a problem, when an identification of single units is desired. Extracellular recordings and long-term electrical stimulations have been performed by means of multi-electrode arrays (Thiébaud et al., 1997; Egert et al., 1998; Duport et al., 1999; Kristensen et al., 2001; van Bergen et al., 2003; Killian et al., 2016), but due to the large electrode pitch and electrode diameter of traditional MEAs, only population activities have been typically observed. To the best of our knowledge, there are no studies showing single-neuron activity that can be continuously tracked over multiple days.

Commonly used organotypic slice cultivation methods include the roller-tube method (Gähwiler, 1981), or the replicated roller-tube method (Egert et al., 1998; van Bergen et al., 2003), the membrane-interface method (Stoppini et al., 1991), and microfluidics-based methods (Eddings and Gale, 2006; Berdichevsky et al., 2009; Rambani et al., 2009; Scott et al., 2013). The principle of the membrane-interface method is the cultivation of slice tissue on a porous membrane at the interface between the culture medium and air (Stoppini et al., 1991). The membrane-interface method offers the advantage of a straightforward implementation, however, for performing MEA recordings, the slice cultures on the membranes need to be flipped so that the neurons can come in direct contact with the electrodes. The contact between the electrode array and the slice cultures can be enhanced by exerting slight pressure on the membrane of the slice culture, but a precise control of this pressure is difficult. If the pressure is not high enough, the contact between the electrode array and slice cultures is not good enough to obtain high-quality recordings of electrical activities of the slice; if the pressure is too high, the cells of the bottom layer, adjacent to the array, can be damaged. Moreover, the membrane slice cultures are usually disposed of after recordings, as the risk for contamination during a recording session is elevated. Therefore, we considered the membrane interface method not to be ideal for realizing long-term slice recordings on MEAs. The microfluidics-based methods and replicated roller-tube method for slice culturing offer the advantage of preserving the cytoarchitecture of the slice cultures, however, these methods ultimately do not yield a monolayer of cells after extended cultivation time (Stoppini et al., 1991). The monolayer structure is very beneficial for 2D MEA recordings, as electrical activities of cells that are in close proximity to the electrodes can be faithfully recorded by means of an electrode array, whereas cells that are further away from the electrode surface are more difficult to record. Moreover, by having a monolayer structure, most, if not all cells of the preparation can be accessed.

In the roller tube method, a slice is attached to a glass cover slip with a collagen gel or a blood clot. Then, the slice is placed inside a roller-tube, which is continuously rotating on a roller drum at low speed. The continuous rotation exposes the slice to both, media and air, each for half a cycle of the rotation; this method provides sufficient oxygen for the slice to survive (Gähwiler, 1981; Gähwiler et al., 1997) while preserving nutrient availability and humidity. We adapted the roller-tube method to cultivate slices directly on a high-density (HD)-MEA microelectronic chip (Frey et al., 2007, 2009), instead of a glass coverslip. Extracellular recording by means of an HD-MEA is non-invasive to neurons and enables high-resolution recordings at multiple time points over weeks (Obien et al., 2015). Additionally, cultivating slices directly on HD-MEAs entails a firm attachment of the slice tissue to the electrode array. This attachment helps to preserve the locations of the neurons in the slice preparation relative to the array electrode positions. Owing to the high-density of electrodes in the array area (3150 electrodes per mm, Frey et al., 2009), the electrical activity of individual neurons can be simultaneously recorded by multiple electrodes (Jäckel et al., 2012; Bakkum et al., 2013). The cultivation of slices directly on HD-MEA chips does not only improve signal-to-noise ratios in the recordings, but also makes it possible to localize and tracked recorded neuronal activities by comparing spike waveforms that have been obtained from multiple electrodes.

**Figure 1:**
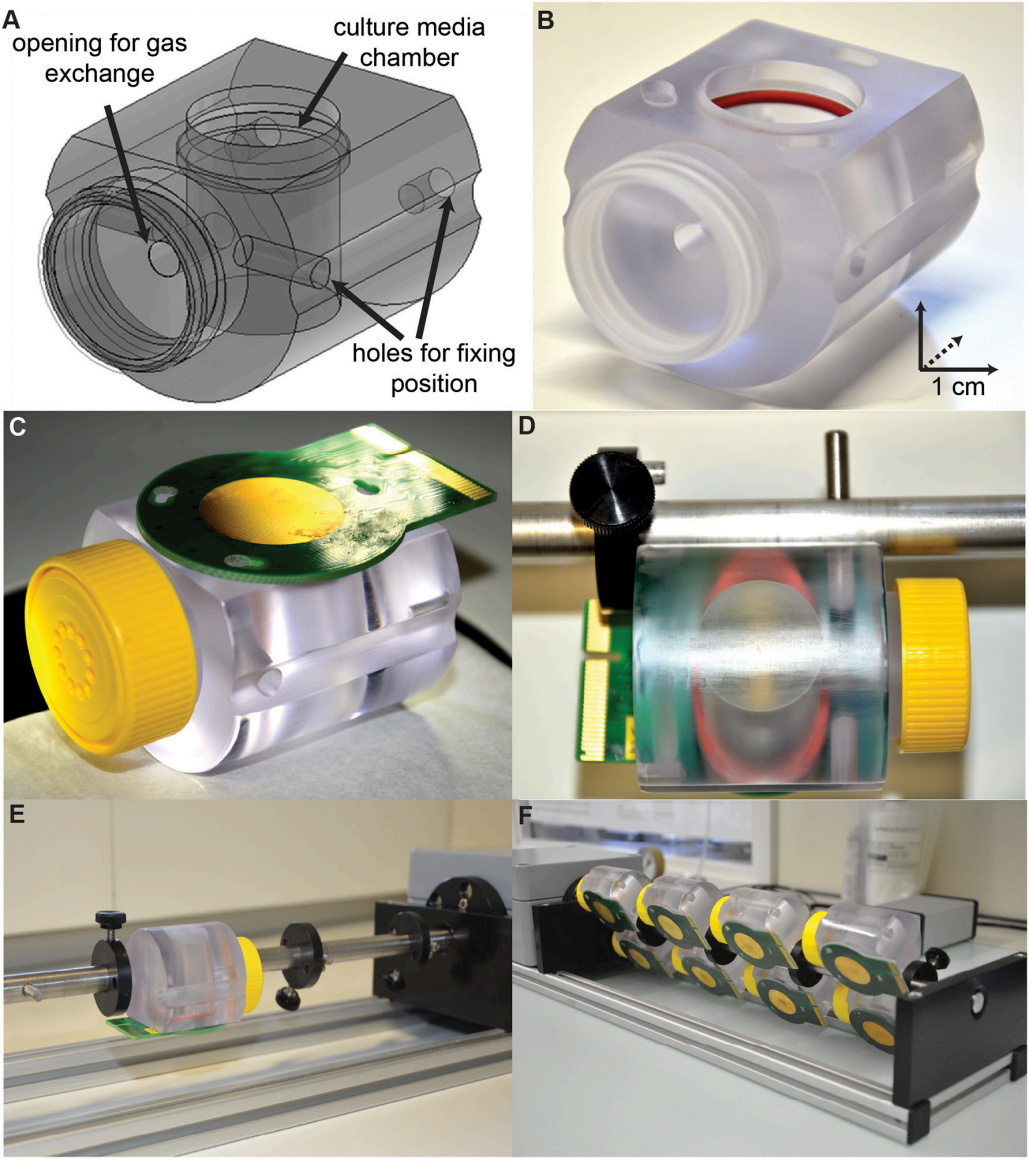
**Setup for culturing organotypic slices directly on HD-MEAs. (A)** A slice cultivation chamber was designed to attach to an HD-MEA chip, hold culture media, and allow for gas exchange. The cultivation chamber was affixed to a custom rotation rack using the two fixing holes. **(B)** The cultivation chamber was equipped with a red rubber O-ring, which was used to form a seal with the plastic ring around the HD-MEA. **(C)** A yellow cap with a sterile filter inside allowed for gas exchange and preserved sterile conditions inside the chamber. **(D)** A cultivation chamber with the HD-MEA attached was affixed onto the rotation rack by using the two holes (one on the side, and the other on the back). **(E,F)** The rotation rack can hold multiple slice cultivation chambers at the same time and provides a continuous low-speed rotation inside an incubator.

In the following sections, we will describe a method for recording spontaneous activity of hippocampal slice cultures by means of HD-MEAs at high spatiotemporal resolution over extended cultivation times. The aim was to track network activity as well as neuronal action potentials at single-cell and sub-cellular resolution. To achieve the goal of recording from the same slice culture at multiple time points over weeks, specific slice culture chambers and rotation racks, which can accommodate the HD-MEAs, were designed (Figure 1). Extracellular recordings with HD-MEAs were performed almost daily. Global network-wide **electrical-activity maps** of the slice cultures were generated based on spike amplitudes detected across all electrodes on the array. Due to the high density of electrodes, the electrical activities of multiple single-units in the slices could be identified and tracked during approximately 1 month. An unsupervised iterative spike-sorting algorithm, based on PCA and k-means clustering, was developed to assign the activities to the single-units. Spike-triggered average extracellular waveforms of an action potential recorded across neighboring electrodes, which we termed a “**footprint**,” were generated. We demonstrated the performance of the method by tracking multiple neurons in three different slice cultures until *day in vitro* (DIV) 23. The locations and magnitudes of peak amplitudes in the footprints and the extensions of the central footprint areas were compared between every two consecutive days. The results showed that the single-unit footprints remained relatively stable over days.

## Materials and methods

### Ethics statement

All cell material acquisition and animal experiments were done in accordance with guidelines approved by the Basel-Stadt veterinary office according to Swiss federal laws on animal welfare (protocol number: 2358).

### Thy1-YFP mice

Thy1-YFP-16 mice (*B6.Cg-Tg (Thy1-YFP) 16Jrs/J*, Feng et al., 2000) were provided by M. Rüegg's lab, Biozentrum, University of Basel, Basel, Switzerland. The Thy1-YFP mice lines were maintained as transgenic heterozygotes by a transgenic × homozygous wild-type (*C57 BL/6*) cross. Genotyping was done through PCR analysis.

### Organotypic hippocampal culture

**Figure 2:**
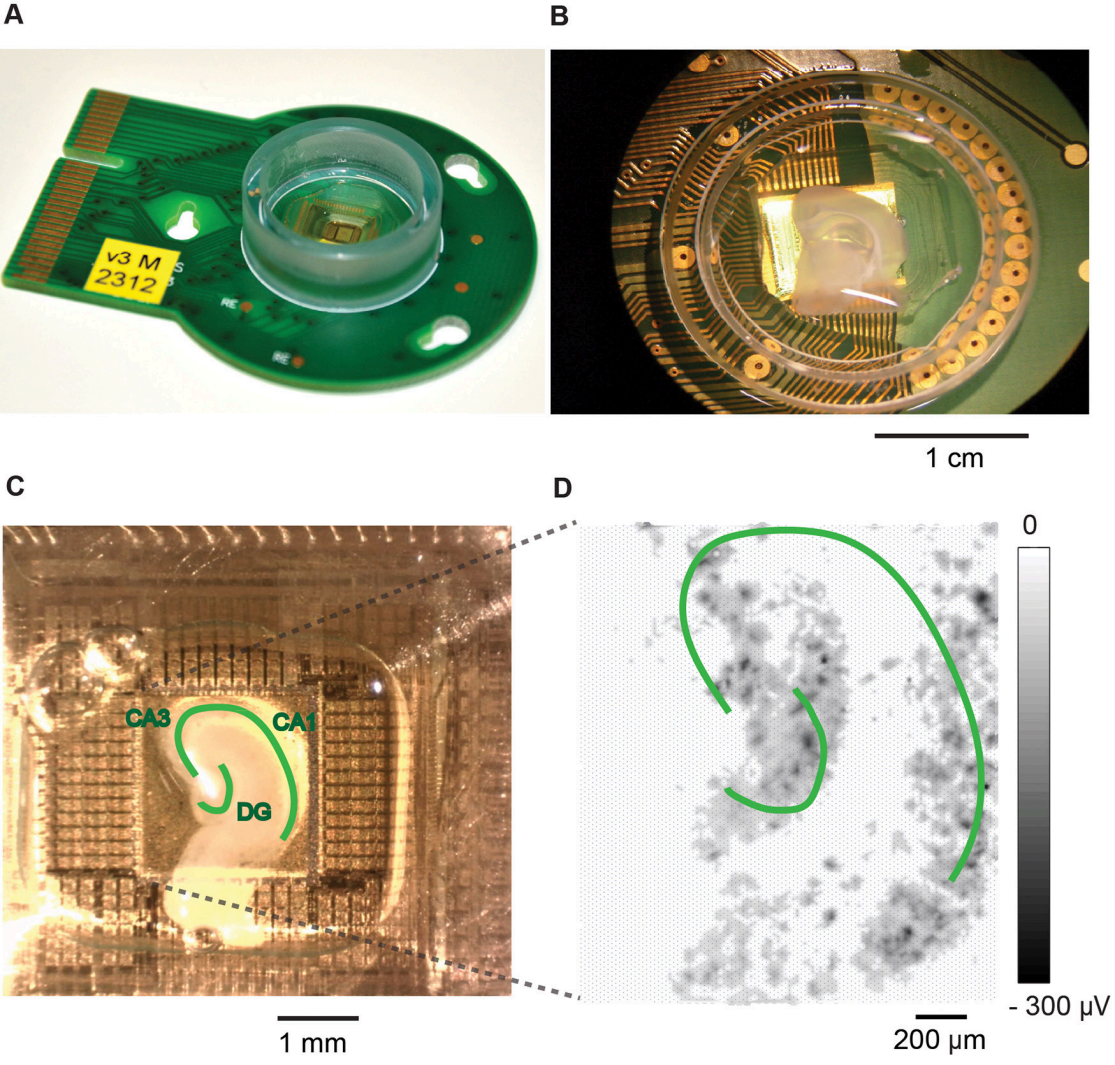
**Hippocampal slice cultivated on and recorded with a HD-MEA. (A)** An HD-MEA chip with a plastic ring and epoxy to encase the bond wires that connect the CMOS chip to the PCB. **(B)** A brain slice on top of the HD-MEA. **(C)** Hippocampal slice attached on the electrode array area with chicken plasma and thrombin. Different sub-structures of the hippocampus are labeled, including the cornu ammonis areas (CA3, CA1), and the dentate gyrus (DG). The photo was taken on DIV 0. **(D)** Amplitude map of the detectable activity from a slice culture. The whole array was scanned to check slice culture activity on DIV 4.

Newborn Thy1-YFP mice, aged between postnatal day 5–7, were used to obtain brain slice cultures. The details of the applied organotypic culturing procedure have been described in previous papers (Gähwiler, 1981; van Bergen et al., 2003). Briefly, brains were removed and placed in ice-cold oxygenated (95% O_2_ + 5% CO_2_) HBSS (HANK's balanced salt solution, GIBCO 14175), mixed with D-Glucose (45%, Sigma G87691), Kynurenic acid (1 mM, Tocris), and penicillin-streptomycin (5 U/ml, GIBCO 15140) under sterile conditions. Bi-lateral hippocampi were dissected and embedded in low-melting-temperature agarose solution (1%, Sigma-Aldrich, A9414). Sagittal hippocampal slices (300 μm thickness) were obtained by using a vibratome (Leica VT1200 S). The HD-MEA chip was sterilized in 70% ethanol for 40 min and coated with 0.05% PEI (polyethyleneimine, pH = 8.5, Sigma-Aldrich; Bakkum et al., 2013) before slice culture attachments. Slices were attached on the HD-MEA surface (Figures 2B,C) by using a mixture of chicken plasma (500 U/ml Sigma-Aldrich P3266) and thrombin from bovine plasma (200 U/ml, Sigma-Aldrich T4648). Photos of the slices on HD-MEAs were taken at this time (DIV 0, Figure 2C). Slice culture medium (3 ml, contained basal medium eagle without L-glutamine, Hanks' balanced salt solution, inactivated horse Serum, 45% D-Glucose, GlutaMAX, with/without penicillin-streptomycin, and with/without B27 supplement) was supplied to each slice culture after the slice culture had been placed on the HD-MEA surface. The hippocampal slices were cultivated in culture chambers (Figures 1A–D), which were kept rotating on a rotation rack (Figures 1E,F), placed inside an incubator with controlled temperature (36°C), humidity (90%) and CO_2_ (5%). Culture medium was replaced after 3 days with culture medium containing penicillin-streptomycin without B27.

### HD-MEA

The complementary-metal-oxide-semiconductor (CMOS)-based, planar, high-density microelectrode array (HD-MEA) was designed and produced in our labs (Frey et al., 2007, Figure 2A). The HD-MEA contains 11,011 platinum electrodes in an array area of 2 × 1.75 mm^2^ with an electrode density of 3150 electrodes/mm^2^. It employs the switch matrix approach to flexibly connect arbitrary subsets of electrodes to the 126 readout channels. The electrode size is 5 × 7 μm^2^, and the center-to-center pitch is 18 μm. Pt electrodes were fabricated on the chip in a post-processing step on wafer-level in the cleanroom. The surface of the HD-MEA chips was afterwards passivated with a stack of alternating SiO_2_ and Si_3_N_4_ layers. The chip was glued and bonded onto a PCB, and the bond wires were encapsulated with epoxy (Epo-Tek 302-3 M). A plastic ring (height: 8 mm, diameter: 2.4 cm, Figure 2A) was added to contain the liquid culturing media. After packaging, a layer of platinum black was electrochemically deposited onto the electrodes in order to reduce the electrode impedance and improve the signal-to-noise ratio. A current of 180 μA was simultaneously applied to all electrodes (current density of 0.5 nA/m^2^) in a solution containing 7 mM hexachloroplatinic acid, 0.3 mM lead acetate, and hydrochloric acid with an adjustment of the solution pH to 1 (Bakkum et al., 2013).

### Electrophysiology recordings

**Figure 3:**
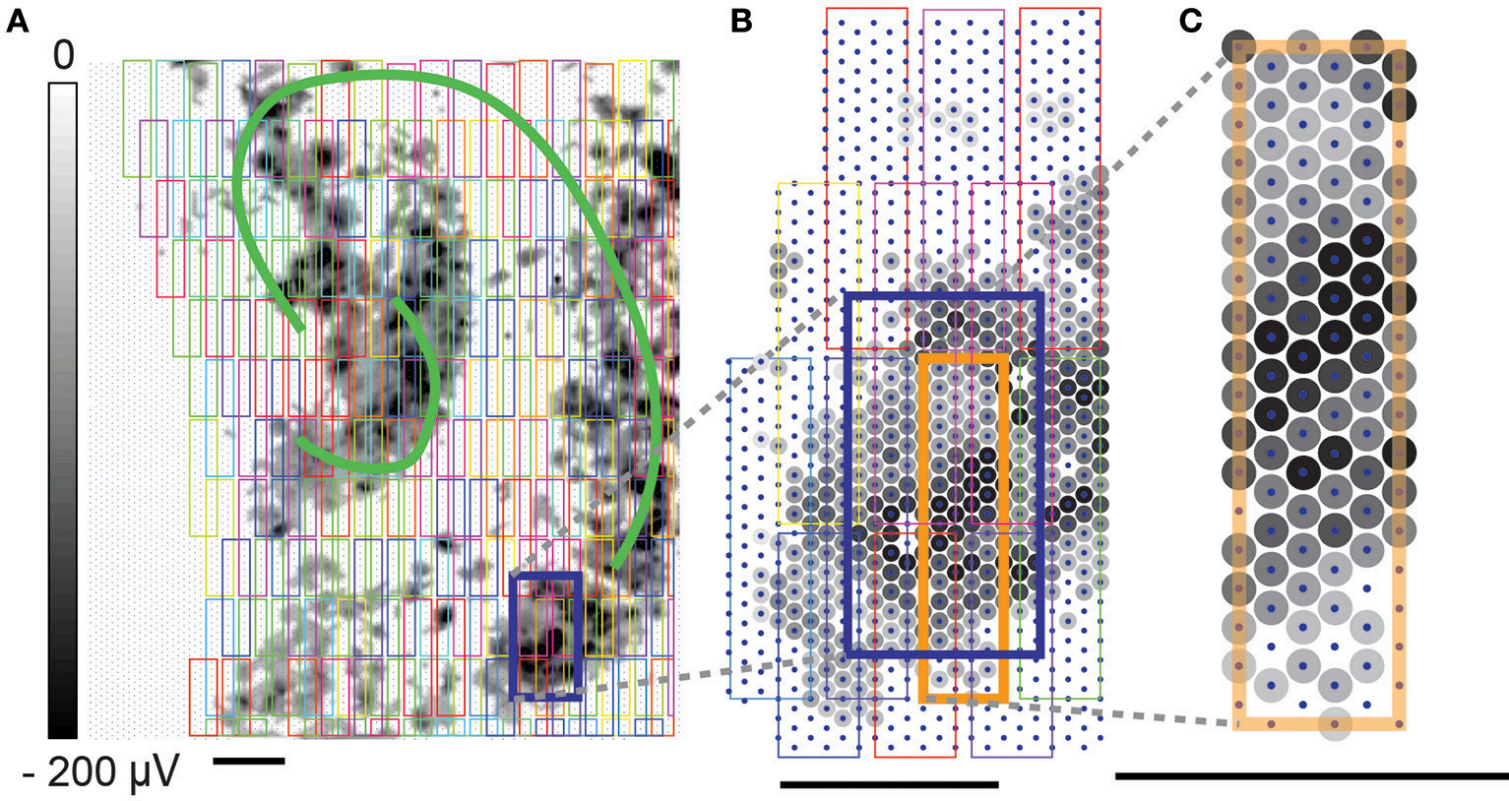
**High-density block configurations**. High-density block configuration recordings were performed for generating global network electrical-activity maps and single-unit footprints. Gray dots indicated the largest negative spike amplitudes detected on the electrodes. **(A)** The electrical-activity map of the slice culture was generated based on the largest negative spike amplitude on each electrode. Slice activity was recorded on DIV4. The cartoon labeling of hippocampus sub-areas was drawn based on a photo on DIV 0. **(B)** Close-up of multiple block configurations (colored rectangles) in a specific recording area (dark blue box). The high-density block configurations were designed with 25% electrode overlap. **(C)** One high-density block configuration contains 18 × 6 (108) recording electrodes. Unlabeled scale bars mark 200 μm.

To perform electrophysiology recordings, a custom-made software, adapted from Meabench (Wagenaar et al., 2005; Bakkum et al., 2013), was installed on a desktop computer. A field-programmable gate array (FPGA) and a microcontroller, embedded in a custom circuit board, were used for data readout from the HD-MEA (Müller et al., 2012). To start a recording session, slice cultures including the culture chambers were removed from the rotation rack and plugged into a stationary setup (Müller et al., 2012; Bakkum et al., 2013) that was also placed inside an incubator with controlled environmental conditions (36°C, 65% humidity, and 5% CO_2_). Before starting experiments, the slices were checked for activity (Figure 2D). Only slices that showed spontaneous activity were included for experiments. Photos of slices taken on the day of plating (DIV 0) and the array-wide activity map were used to identify the area that the slice occupied on the HD-MEA. The slice area showing activity was then recorded with a series of high-density block-shaped configurations. To generate these serials of configurations, a boundary line was identified along the edge of the area that the slice occupied on the array. The total number of electrodes within this area was calculated, and multiple block-shape electrode groups were generated (block size: 6 × 18 electrodes, which covered an area of approximately 80 × 320 μm^2^, Figure 3C), with 25% overlap between the electrode blocks (Figures 3A,B). To assign the electrode blocks, we started with the first block at the upper left corner of the selected area, and then generated the next adjacent block (smaller value on y axis, larger value on x axis) by also including 25% of the electrodes of the previous block that were closest to the new block. Each block was recorded during 40 s, so that one recording session lasted about 1.5 h. Recordings were performed almost every day between DIV 6–30 to record the network activity and single-unit activities of the slice cultures. A closed-loop controlled cooling device (built in house) was attached to the HD-MEA to maintain a stable temperature (36°C) during recordings. Before each recording experiment, slices were left alone for 30 min to allow for habituation.

### Data analysis

To extract extracellular action potentials, the signals recorded on the HD-MEA were sampled at 20 kHz, filtered with a digital band-pass (500–3000 Hz), and a threshold detection was applied (5.5 times the noise standard deviation). A MATLAB (R2012a and R2014b) code was used for data analysis (Jäckel et al., 2012; Müller et al., 2012).

#### Global network activity

The global network activity of the slice cultures was evaluated with two different methods: (1) by using the “electrical-activity map” without spike sorting; (2) by assessing the total number of neurons that could be detected through spike sorting of the high-density block recordings. To generate the electrical-activity map of the slice culture, the largest absolute values of the negative amplitudes of the extracellular action potentials on each electrode were plotted (see also **Figure 6** later in the article).

#### Spike sorting and single-unit footprint generation

**Figure 4:**
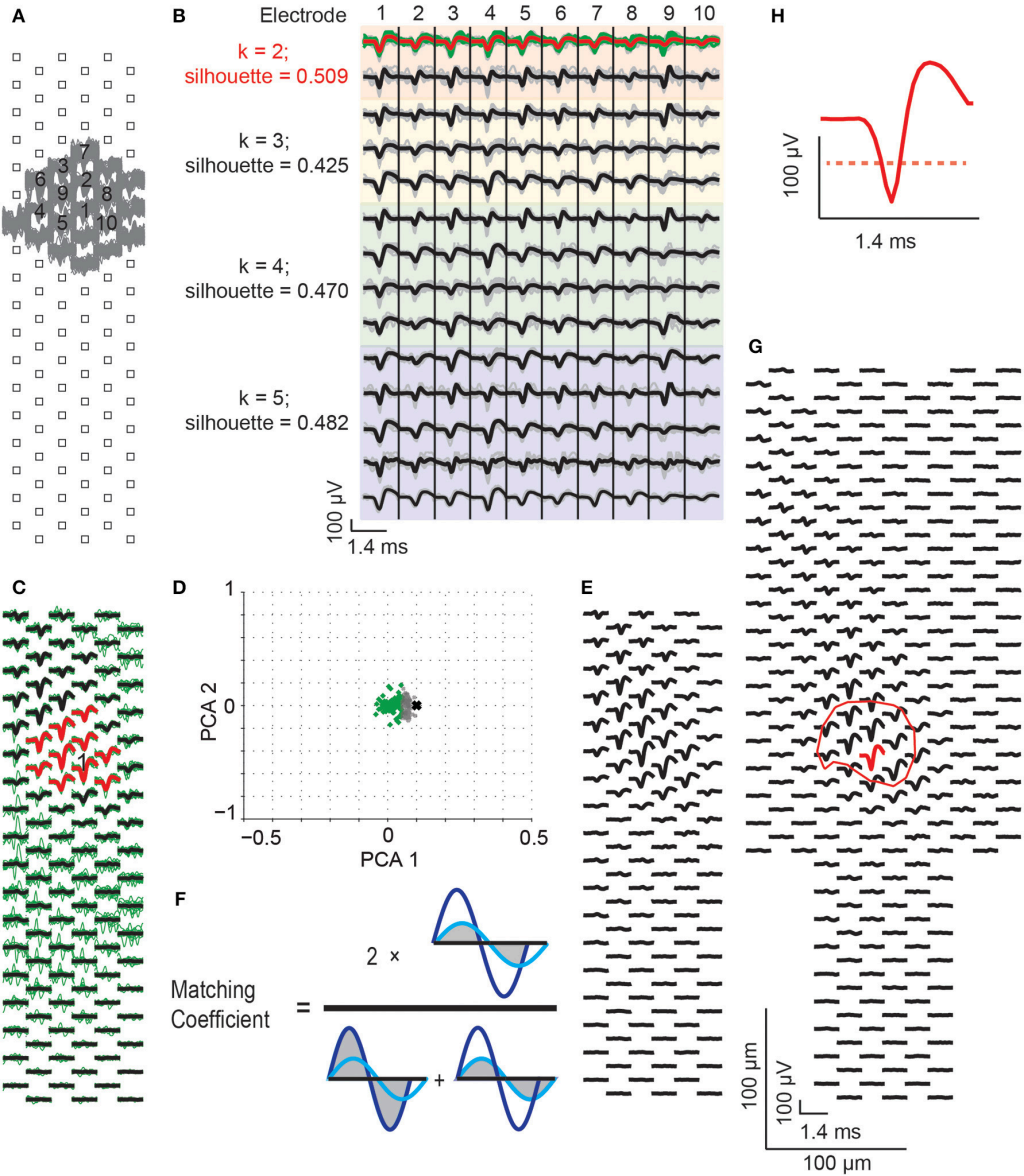
**Spike sorting procedure. (A)** Raw spikes (gray traces), recorded from a block configuration, were identified via threshold detection. The electrode with the largest spikes was selected as the “core electrode” (labeled as electrode “1”), and the neighboring electrodes having the largest spikes were identified (gray traces). From all these electrodes, the 10 electrodes with the largest spikes were chosen for spike sorting. **(B)** Spike waveforms from the selected 10 electrodes were up-sampled by a factor of 6 in time (gray traces, median value in black). The waveforms were clustered with PCA and *k*-means while using different numbers of clusters (*k* = 2, 3, 4, and 5, in the colored panels). The silhouette scores were assessed to detect the best number of clusters to use, which was indicated by the largest silhouette values (here, the orange colored panels featuring 2 clusters). The selected cluster (green traces), with the largest spike at the core electrode, was chosen for generating the spike template (the median of the green traces, indicated as red trace). **(C)** From spikes in the selected cluster, a spike template was generated covering all electrodes in the respective configuration. The green traces are the waveforms that belong to the sorted neuron. Black and red traces are the spike template for this neuron, which is the median of the green traces. The red traces show the template for the 10 chosen electrodes in **(A)**, and the core electrode is labeled with “1”. **(D)** All recorded spikes from the selected 10 electrodes were subtracted from the spike template to calculate residuals, in order to avoid the over-sorting issue from the spike-clustering step. The residuals were, again, clustered by PCA and k-means (*k* = 2). Green dots are the residuals that match the template, gray dots are the residuals that do not match the template. The black cross represents the negative template signals (i.e., zero minus the template, which is used to represent the location of a signal with only noise in the PCA space). **(E)** The spike template was re-calculated from the mean signal of the spikes matching the template (i.e., the spikes that generated the green dots in **D**). **(F)** The generated spike templates, termed “single-unit footprints,” were compared between overlapping configurations and within individual configurations in order to merge templates arising from the same neuron. This was done by calculating the matching coefficients for waveforms at all common configuration electrodes. The matching coefficient was calculated as 2 times the overlap area between the dark and light blue waveforms, divided by the sum of the two individual waveform areas. Areas are indicated with gray color. Based on the matching coefficient values, different templates were merged (see text for more details). **(G)** Final template of a sorted neuron across multiple configurations. Red is the core electrode, where the *CF amplitude* is determined. The respective electrode location defines the *CF location*. The red circle indicates the core area of the single-unit footprint, which was defined as *CF area*. **(H)** A zoom-in of the spike at the core electrode. The red dashed line marks the threshold of 50% of the largest negative signal amplitude within the footprint area, which has been applied to calculate the *CF area*. The scale bars in **(G)** also apply to **(A,C,F)**.

To extract single-unit activities, off-line spike sorting was performed with an automated spike-sorting analysis on each recording configuration. The footprints of single units were generated through spike clustering and template matching steps, and template merging steps were used to deal with neurons that extended over adjacent configurations. The spike sorting steps are described as follows:

Step I. Spike clustering:

- Spikes were extracted via threshold detection (5.5 standard deviations above the noise level) for each of the recording electrodes.
- Negative spike amplitudes were ranked for each electrode. The electrode with the largest spikes was selected as a “core electrode” (Figure 4A).
- As neuronal action potentials could be detected on several neighboring electrodes simultaneously, the spike times detected from the core electrode were used to investigate recordings of neighboring electrodes (Figure 4A).
- From these, the 10 electrodes recording the largest signals were selected to assist spike sorting.
- The spike waveforms of the selected 10 electrodes were up-sampled by a factor of 6 in time. Spike clustering was done via PCA and *k*-means (Lewicki, 1998; Duda et al., 2001) with *k* = 2, 3, 4, and 5, where *k* is the number of clusters. The silhouette analysis was then applied to determine the optimal number of clusters to use (Rousseeuw, 1987; Sadgrove et al., 2006). The *silhouette coefficients* vary between -1 and 1, which respectively indicates “misclassified” and “well-clustered” data. The following analysis was done for every *k*: (i) a *silhouette value* was obtained for each spike waveform to assess how well each spike belongs to its cluster. (ii) The mean of all *silhouette values* was computed. The cluster with the largest mean of *silhouette values* was chosen (Figure 4B). If the spike clusters had *silhouette values* larger than 0.5, and the biggest spikes from these spike clusters were larger than 80% of the largest spike amplitude, these clusters were further considered as candidates for generating spike templates (Figure 4B).
- The spike times of one selected spike cluster were used to detect spikes from all other recording electrodes in the configuration. The median values of spikes, identified as belonging to the sorted neuron, were calculated for each recording electrode and generated the spike template in Figure 4C.

Step II. Template matching:

- To confirm that all recorded spikes belonging to the sorted neuron had been collected from the selected 10 electrodes, and to avoid the same neuron being sorted as two different neurons due to over-sorting at the spike-clustering step, all recorded spikes from the 10 electrodes were compared again with the sorted neuron template by temporarily subtracting all spikes from the template. The residuals were sorted into 2 new clusters via PCA and *k*-means (with *k* = 2, Figure 4D, the green and gray dots). The residual clusters were evaluated based on relative values of the first PCA component. If one cluster was at least two times closer to the origin of the first PCA component than the other cluster, then the first cluster was recognized as belonging to the sorted neuron. Otherwise, both of the two clusters were considered as coming from the same neuron. As a result, all the spikes that featured similar waveforms as the template were collected at this step and were included to produce the *single-unit footprint* (Figure 4E). Until this point, spike sorting was done for one neuron in one configuration. Thus, a *single-unit footprint* represents the spatiotemporal extracellular waveforms of a single neuron, which has been calculated by spike-triggered averaging of signals according to the spike times at the electrode with the largest signal.
- To sort the second neuron from the same block recording, the spikes from the first sorted neuron were permanently subtracted from the recordings, and steps I (a) to II (a) were repeated.
- Single-unit footprints were established for all recording blocks over the whole array.

Step III. Merging templates within and across configurations:

To assess whether the obtained single-unit footprints originated from the same neuron, the spike waveforms of all detected single-unit footprints were compared, and a matching coefficient value (Figure 4F) was calculated for every possible pair of single-unit footprints. When comparing across configurations, only electrodes common to both configurations were considered. The single-unit footprints recorded from the same recording block were considered as belonging to the same neuron if they had matching coefficients larger than 0.95. The single-unit footprints, recorded from adjacent recording blocks, were considered as belonging to the same neuron if they had matching coefficients larger than 0.85. In case of large enough matching coefficients, the single-unit footprints were merged (Figure 4G). The total number of detectable single-units in a slice culture was measured at each day of the recordings and was used as a second method to analyze the global network activity of the slice culture.

#### Single-unit footprint tracking

**Figure 5:**
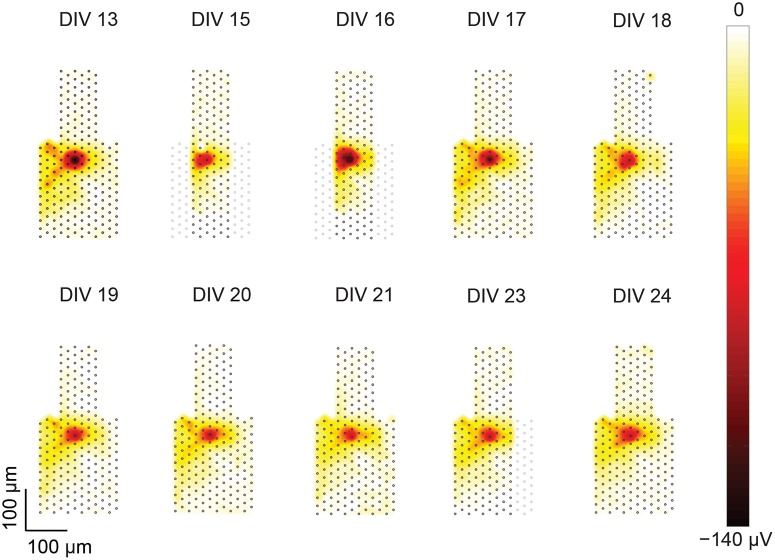
**Heat maps of a single-unit footprint tracked over multiple days**. Footprints of a single unit from DIV 13–24 plotted as heat maps. The positions of the electrodes within the included recording blocks are represented as gray dots. For DIVs 15, 16, and 23 some of the electrodes are missing due to a recording artifact as described in the text. The shape of the central footprint area (red), however, was intact and remained stable over the recording duration.

The matching-coefficient method (Figure 4F) was also used for tracking single-unit footprints recorded on different days. Single-unit footprints generated from the same block configurations were compared between consecutive recording days. If they had matching coefficients larger than 0.85, the respective single-unit footprints recorded on different days were considered as belonging to the same neuron. Single-unit footprint tracking was automatically done with a software (MATLAB), and the results were checked and evaluated manually. Only neurons showing traceable activity during at least 7 recording days were included in the manual evaluation (an example is shown in Figure 5).

The single-unit footprints tracked on different days were presented as heat maps (Figure 5). Single-unit footprints that belonged to the same neuron, showed similar shapes on the heat maps over different days. Manual evaluation of the single-unit footprint tracking was performed by comparing the heat maps of the single-unit footprints across different days. Data that met the following criteria were then manually removed: (1) single-unit footprints with different heat map shapes that were mistakenly recognized as identical neurons by the software; (2) single-unit footprints that had negative peak footprint amplitudes of less than 50 μV (i.e., their amplitudes were too close to noise levels); (3) single-unit footprints that showed multiple footprint centers on heat maps; (4) the central footprint area of single-unit footprints which was partially missing.

Missing parts of a single-unit footprint can be an artifact of the recording procedure, in which not all electrode blocks were recorded at precisely the same time. Missing neuron parts could occur, if the neuron had not been spontaneously active while the respective electrode block (in which the neuron or parts of the neuron were located) was recorded. Then, upon merging the electrode blocks, the respective neuron part would be missing. The consequences of this recording artifact can, for instance, be seen in Figure 5 in the heat maps of DIV 15–16 or 23, where a fraction of the recording area is missing. But in this case, the central footprint area was intact, therefore, this neuron could still be included in the tracking analysis.

#### Single-unit footprint dynamics analysis over days

The largest negative spike within the footprint was allocated (Figure 4G), and the absolute value of the negative peak amplitude was defined as the **central footprint peak amplitude (*CF amplitude*)**. The precise locations of the peak spike with regard to the electrode positions was calculated by using cubic interpolation and a spatial up-sampling by a factor of 100, which yielded a spatial resolution of 2 μm over the array. The location of the largest amplitude-signal within the footprint was used to define the **central footprint location** (***CF location***). A threshold of 50% of the largest-amplitude signal (red dashed line in Figure 4H) was used to defined the **central footprint area** (***CF area***) (Figure 4G, red circles), which included electrodes that had negative spike amplitudes larger than 50% of the *CF amplitude*. The *CF location, CF amplitude*, and *CF area* values of the tracked single-unit footprints were calculated for each recording day.

The distance that the *CF location* moved between consecutive recording days (*di*) was compared, and calculated as:

$$\begin{array}{l} Movement\;distance\;\left(di\right)=abs\;(CF\;location\;(di)\\ \;-\;CF\;location\;(di-1)) \end{array}$$

The group medians were compared across days for the *CF location* movement analysis. The group mean was used for the *CF amplitude* analysis and the *CF area* analysis. In total, data from 22 single-units tracked in 3 different cultures between DIV 16–21 were used to analyze the single-unit footprint dynamics.

## Results

### Roller-tube system

To observe the changes in electrical activities of an organotypic slice culture with HD-MEA recordings over extended time periods, a roller-tube setup, adapted to the HD-MEA format, was developed in house. This system allowed for culturing brain slices on HD-MEAs over weeks, while HD-MEA electrophysiology recordings could be performed at any time during the cultivation period. The roller-system consisted of culture chambers and a rotation rack (Figure 1). The culture chamber included a culture media chamber, an opening for gas exchange, and holes for positioning. A rubber ring (Figure 1B) was inserted into the culture media chamber to form a seal with the plastic ring surrounding the HD-MEA (Figures 1C, 2A), which created an enclosed chamber space that preserved culture media. A cap with a sterile filter (Figure 1C) allowed for gas exchange between the culture chamber and the incubator environment. The culture chamber was fixed on the roller rack at two fixing holes, one on the side of the culture chamber and one on the other end of the chamber (Figures 1A,D). The roller rack was designed to continuously rotate at a very slow speed (about 1.5 min per cycle). Each roller rack held a maximum of 8 culture chambers with HD-MEAs, and was placed inside an incubator with controlled temperature (36°C), humidity (90%), and CO_2_ level (5%).

The results evidenced successful and reliable culturing and recording of organotypic slice cultures on HD-MEAs with the roller-tube method. In total, 32 slice cultures from 7 different preparations were produced. Of these, 26 slice cultures showed spontaneous activity on the first day of HD-MEA recordings. There were 20 cultures (out of the 26 cultures) still showing activity after DIV 10, 12 cultures after DIV 20, and 4 cultures after DIV 30. Five cultures from 2 different preparations were recorded from consistently between DIV 13–23, and these data were used to generate electrical-activity maps and to perform single-unit detection for network analysis. Three of these five cultures were selected for single-unit tracking over days.

### Global network activity of slice cultures over days

**Figure 6:**
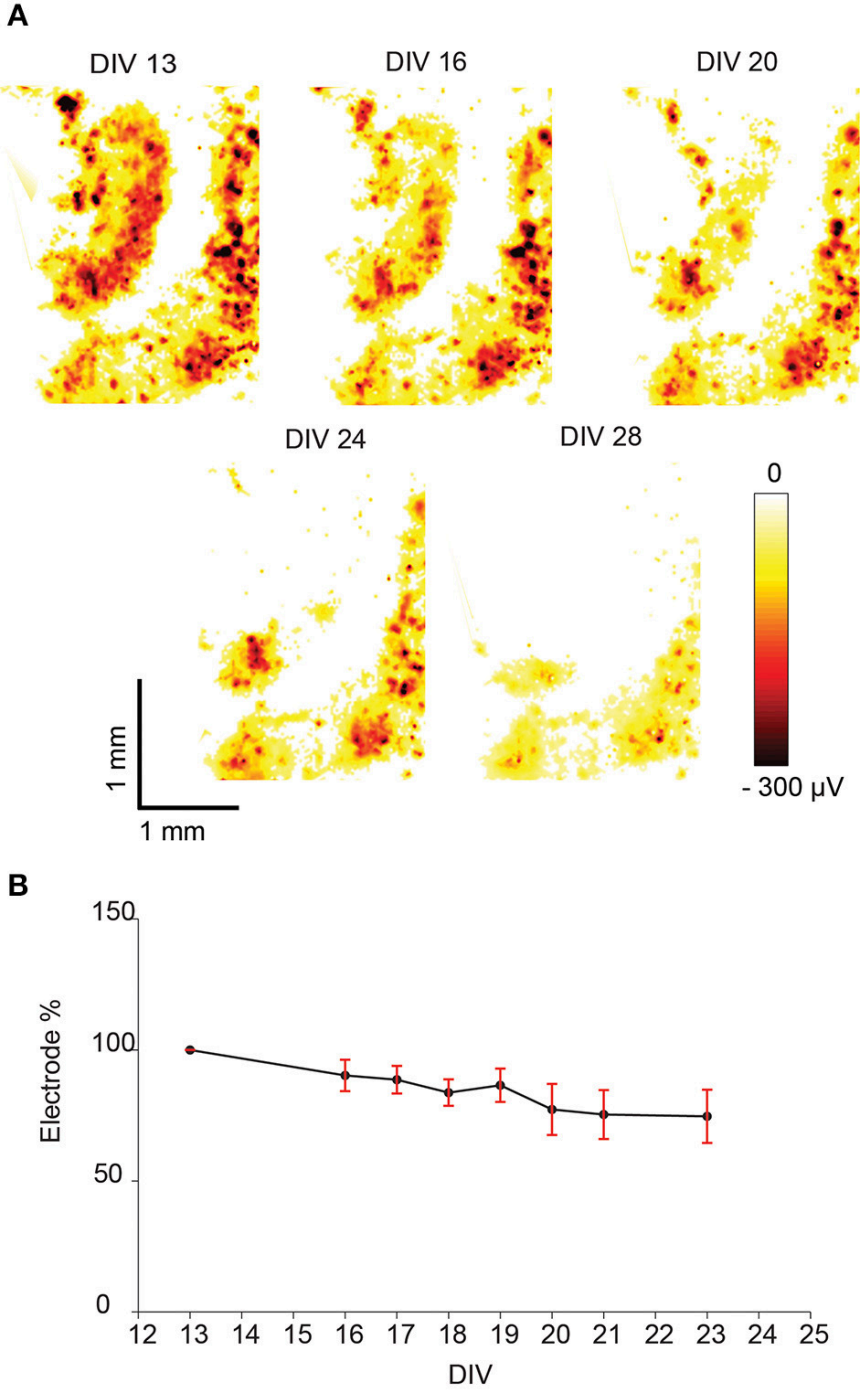
**Spontaneous activity of a hippocampal slice recorded with an HD-MEA during several days and displayed as “activity map.” (A)** Example of spontaneous activity in a hippocampal slice culture observed over several days. The largest negative spike amplitudes produced by spontaneous activity are displayed as a heat map across the whole array, dark red color indicates the largest negative spike amplitude. **(B)** The percentage of electrodes that detected spikes exceeding the amplitude detection threshold (mean ± SEM, *n* = 5). The percentage was normalized to the number of electrodes detecting activity on the first day (100%). In all cases the percentage decreased with increasing cultivation time.

As expected, the global network activities of the slice cultures decreased while increasing cultivation time. Electrical-activity maps were generated for each slice culture during every recording session, based on the largest absolute signal amplitude (larger than 50 μV) extracted from the spikes on each electrode (Figure 6A). The number of electrodes that still detected activity was counted at each day of recording, and was normalized with respect to the first day of recording. The relative percentage of electrodes that detected activity decreased in average by 25% from DIV 13–23 (Figure 6B).

### Single-unit activity obtained from spike sorting

**Figure 7:**
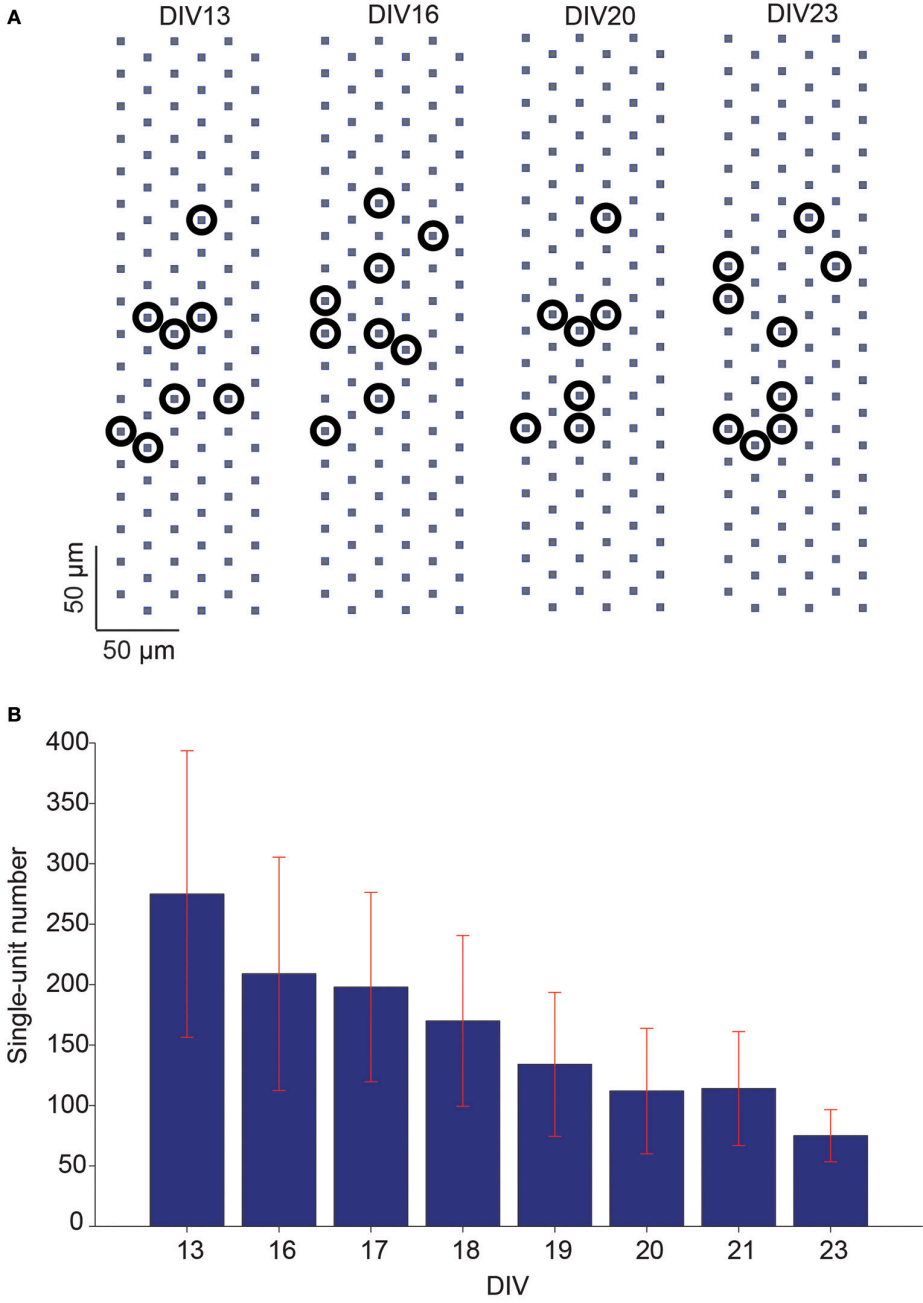
**Number of single-units detected in slice spontaneous network activity over days. (A)** Example of multiple single-units, identified via spike sorting from the same recording block configuration, on different recording days. Due to the high electrode density, the locations of identified single-units could be assigned according to the electrode positions. The approximated locations of the detected single-units are represented by black circles. **(B)** The mean of number of detected single-units decreased over days in a set of hippocampal slice cultures (mean ± SEM, *n* = 5).

The overall network activity of the slice cultures was also characterized in terms of detectable single-units at each recording day. Due to the high electrode density of the HD-MEA, single-unit activities could be identified from the high-density-block recordings with the help of an automatic spike sorting software developed in house. Multiple single-units could be identified within the same high-density recording block, and each single-unit location could be defined according to its nearby electrode positions (Figure 7A).The number of detected single-units was counted on each recording day and normalized with respect to the number on the first day of the analysis (DIV 13). The number of detected single-units decreased in average by 72.7% between DIVs 13–23 (Figure 7B).

### Single-unit-activity tracking over days

**Figure 8:**
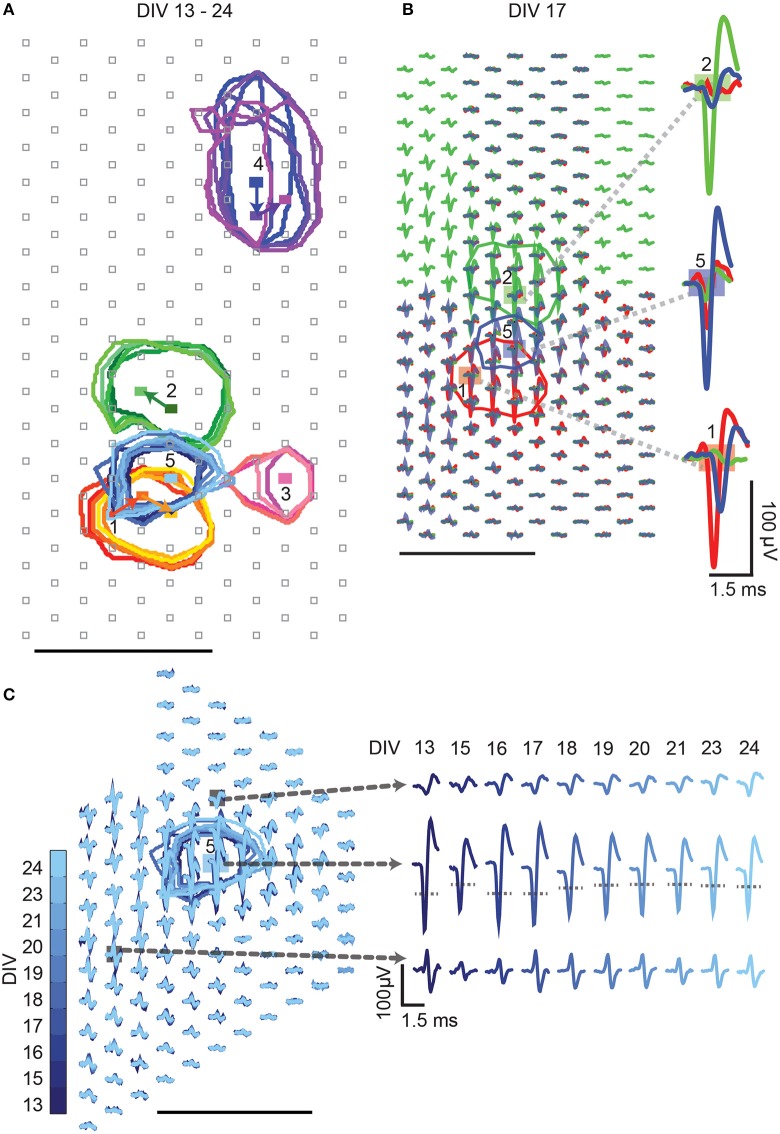
**High-density block recording analysis**. Activities of multiple single-units can be identified from high-density block recordings over multiple days. Different colors represent the different single units, which are labeled with numbers. Color shades indicate the different recording days: darker colors indicate early DIVs, lighter colors indicate later DIVs. The colored circles mark the CFA of the respective single units. Rectangles indicate the CFs. Gray squares represent electrode locations. **(A)** Multiple single-unit activities were distinguished and tracked over different days. The movements of the CFs over days are indicated by the colored arrows. **(B)** Detailed waveforms of three overlapping single-unit activities (1, 2, and 5 from **A**) recorded on DIV17 are shown. The spike waveforms of the three single-unit activities can be clearly distinguished based on spike shapes and amplitudes. Examples of waveform separations at the respective CF locations are shown. **(C)** Tracking of single-unit activities over days. The CFAs of single-unit # 5 are, again, represented by blue circles. Waveforms of single-unit # 5 at three different locations are shown over days, and the CF location is marked by the blue rectangle. The gray dotted lines in the waveform plots indicate the 50% amplitude threshold. Unlabeled scale bars mark 100 μm.

**Figure 9:**
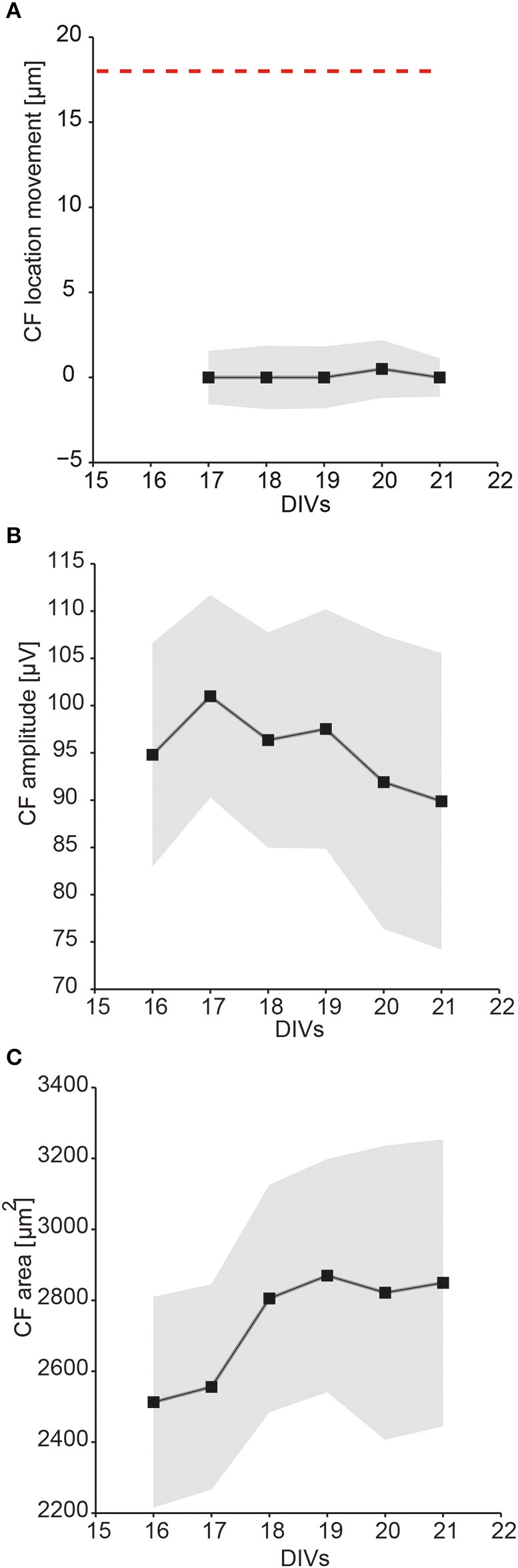
**Statistics on multiple single-unit activities over days**. The tracked single-unit footprint patterns were characterized according to their *CF location, CF amplitude*, and *CF area* values. The average distance that the *CF location* moved, the average *CF amplitudes*, and the average *CF area* sizes were calculated based on 22 neurons from three different cultures between DIV 16 and 21. The average values of all tracked single-units are displayed as black squares, the corresponding errors as gray-shaded areas. **(A)** Distance that single-unit *CF locations* have moved between two consecutive recording days (median ± SEM). The red dashed line indicates the electrode pitch of 18 μm. **(B)** The *CF amplitudes* over days (mean ± SEM). **(C)** The *CF area* size over days (mean ± SEM).

In a next step, we wanted to demonstrate that activities of multiple single-units can be identified (Figure 8A), separated (Figure 8B), and tracked (Figure 8C) within the same slice cultures over multiple recording days (Figures 8, 9). The single-unit footprint patterns were characterized according to their electrical-footprint *CF location*, the *CF amplitude*, and the *CF area*. The *CF locations* were compared between every two consecutive recording days (calculations provided in the *Materials and Methods* part). The analysis was performed on 22 neurons, which were tracked between DIV 16–21.

The results showed that single-unit activity patterns in the slice cultures changed over time, but that the magnitudes of the changes between every two consecutive recording days were relatively small. Between DIV 16–21, the average distance of the *CF location* movement was close to zero (Figure 9A). The footprint *CF amplitude* magnitudes varied from neuron to neuron, were about 100 μV on average, and slightly decreased over days (Figure 9B). The *CF area* sizes varied from neuron to neuron, on average about 2200 ~ 3200 μm^2^, and increased over days (Figure 9C).

## Discussion

This study presents a new method to continuously characterize neural networks and single-unit activity of organotypic hippocampal slices over long periods of time. Compared to dissociated cell culture preparations, organotypic brain slice cultures largely preserve the neuronal network architectures and cell type combinations, so that they are considered more representative of functional brain areas (Gähwiler, 1981; Stoppini et al., 1991; Bahr, 1995; Gähwiler et al., 1997; De Paola et al., 2003; De Simoni and Yu, 2006; Galimberti et al., 2006). Due to the relatively high cell density in slices (over 10,000 cells/mm^2^, Abusaad et al., 1999; Sadgrove et al., 2006), slice cultures pose larger challenges to single-unit activity identification and long-term tracking of the very same neurons in comparison to cultures of dissociated neurons. Although, single-neuron electrophysiological studies in slices are commonly performed by using the patch-clamp technique, this method is invasive, and, therefore, only suitable for one-time experiments per culture, so that chronic studies can hardly be performed.

To fill this gap, we developed a setup, which allows for cultivating organotypic slices directly on HD-MEAs. High spatiotemporal-resolution recordings allow for tracking single-unit activity over extended periods of time. At the same time, network activities can be studied, and the measurement time window is flexible. Slice cultures can be efficiently used for a sequence of experiments, large numbers of single-units per recording session can be found and studied, and a tracking of single-units over multiple days is possible. This holds particularly true, as the registration of the slice and its units with respect to the array electrodes is largely preserved over the entire culturing and recording time due to the firm attachment of the slice to the chip surface. This is an advantage in comparison to other slice cultivation methods, for instance the membrane interface method (Stoppini et al., 1991), which requires flipping the slice culture upside down to bring the tissue side in contact with the recording electrodes, so that the application of marking on the slices is necessary for knowing its orientation. Moreover, repeatedly and reproducibly placing a slice with large precision on the electrode array poses a major challenge, slices are easily damaged by being pressed down to the electrode surface with an extra weight.

Besides the preservation of the relative position of slices and electrode array in repeated measurements, the cultivation of slices directly on the array provides better contact between slice tissue and electrodes, which improves the recording signal quality. There is no need for marking of the slices. A photo can be taken after the slice plating on the HD-MEA, and the photo can then be aligned—according to the visible electrode positions—with the electrical activity readings of the slice network or single units. Here, we showed that the electrical activity maps could be aligned with photos of the slices to identify the relative position of the hippocampal architecture on the microelectrode array.

In comparison to other recording methods, HD-MEA recording is non-invasive to neurons, and, therefore, does not impact basic cellular physiology. Moreover, there are no problems or limitations associated with phototoxicity effects and bleaching, or limited recording times, which are common for many optical methods.

On the other hand, there are some open issues that became evident in this study. For instance, although spontaneous activities of hundreds of neurons could be recorded in a single hippocampus slice culture, there were many silent neurons that did not show any activity, or alternated between active and silent states during different recording sessions. These silent but not active neurons could be identified through immunohistochemical staining. This also aggravated the problem of optically identifying the recorded neurons, as all viable neurons were immunohistochemically stained after fixing the slice. Furthermore, slice cultures take about one to 2 weeks to thin down to about 1–2 cell layers (Humpel, 2015), but there are multiple cell layers in the slices at the beginning of the cultivation period. Signals from neurons located further away from the electrodes contributed to background electrical activity or “noise” during early DIVs.

To observe the electrical activity patterns of slices during cultivation, electrical-activity map recordings and spike-sorting based single-unit detections were performed between DIV 6–30. A more detailed neuron-tracking analysis was performed for the three cultures that were consistently recorded between DIV 13–23. According to previous studies, the optimal experimental time window is DIV 6–18, and slice viability decreases after DIV 18 (Nagerl et al., 2004; De Simoni and Yu, 2006; Galimberti et al., 2006). Investigations of pyramidal neuron spine electrophysiology and density in organotypic hippocampal slices showed that they were comparable to those in acute slices at equivalent stages (Boyer et al., 1998; McKinney et al., 1999; De Simoni et al., 2003). Our electrical-activity maps of slice cultures were based on detecting the amplitudes of spontaneous spiking activity across all electrodes on the HD-MEAs. The electrical-activity maps evidenced that a large fraction of the overall slice area showed large spike amplitudes. Besides gradual decreases in slice network activity and single-unit activity partially caused by cell death (Stoppini et al., 1993), and besides temporal silence periods in neuron activity fluctuations, neuronal activity levels also were impacted by media changes or pH- and temperature variations (Su et al., 2011; Mewes et al., 2012). Any decreases in spike amplitude will entail difficulties in signal detection and assignment of the signals to the respective single-units.

The spike-sorting based analysis provided more detailed information on the number of electrically active single-units in the slice network. Most cell number analyses for organotypic slices were performed through staining and imaging, in previous studies (Abusaad et al., 1999; Guldimann et al., 2012), and showed that the cell viability remained at 60% in hippocampal slice cultures between DIV 0 and DIV 49 (Guldimann et al., 2012). However, due to the existence of silent neurons that then also had been stained, the imaging of fixed cultures may potentially overestimate network activity in slice cultures. On the other hand, the number of single-units that can be sorted from extracellular recordings depends on many issues, including the length of the recording time, and the signal-to-noise ratio. Prolonged recording periods provide the opportunity to obtain a larger number of spikes, which yields higher spike-sorting precision. However, as the brain slice is quite thick, especially at the beginning of the cultivation period, the overall recording durations should not be excessively long, to not promote cell death through hypoxia. Moreover, decreases in spike amplitudes have been observed toward the end of the daily recording sessions in case that devices to control the array temperature were not used.

To demonstrate that the activity of the same single unit can be continually observed across multiple days with the method presented here, the single-unit activities as detected on each day were compared and tracked by spike waveform comparisons. The spike waveform analysis over days has been demonstrated in other studies (Rousche and Normann, 1998; Schmitzer-Torbert and Redish, 2004; Guldimann et al., 2012), and the authors argued that very similar waveforms can be used to infer that the same neuron has been recorded from. In our measurements, single-unit footprints were observed to be relatively stable over the recording duration. We are therefore convinced that it is possible to investigate the activity or the extracellular action potentials of the same neurons in slice cultures over a longer time, and that extracellular neural activity footprints can be used to identify single-unit neurons.

## Conclusion and outlook

We developed a method to culture organotypic hippocampal slices on HD-MEAs, with the goal to study hippocampal network dynamics and track single neuron activity during the early stages of brain development. The established method offers the potential to study chronic impacts of drugs or genetic modifications on individual neurons in slice preparations. Single-unit activity can be tracked over extended periods, which may become important for neuron implantation studies and single-unit pharmacological studies in the future. Further improvements to the current method will deal with the issues we encountered in this work. A main development thrust includes increasing the overall number of simultaneously readable electrodes, which then yields larger blocks and less required total experiment time and less artifacts in the footprints. An 8-fold increase in the number of simultaneously readable electrodes has been recently achieved (Müller et al., 2015). This will also allow to increase the recording time for each high-density configuration in order to obtain more data useful for single neuron tracking over days. To counteract hypoxia and medium depletion during the recording sessions without device rotation, a microfluidic system for perfusion of oxygenated media will be developed. Moreover, electrical stimulation experiments (Bakkum et al., 2013) can be performed, to identify and possibly activate silent neurons and to investigate axonal signal propagations and neural network plasticity.

## Author contributions

WG performed all experiments and wrote the manuscript. JS developed the software for spike sorting and single-unit activity tracking. DB supervised the experiments and edited the manuscript. DJ developed MATLAB scripts to record network activity and contributed to project discussions. MO assisted in writing and contributed to discussions. MR contributed to figures and the discussions. AH contributed to the design of the roller tube setup, project discussions, and edited the manuscript.

## Funding

This project was financially supported by the EU Marie Curie Initial Training Network (ITN) EngCaBra Contract No. 264417, and the European Research Council Advanced Grants 267351 “NeuroCMOS” (FP7) and 694829 “neuroXscales” (Horizon 2020), as well as the Swiss National Science Foundation through Grant 205321_157092/1. The funders had no role in study design, data collection and analysis, decision to publish, or preparation of the manuscript.

### Conflict of interest statement

The authors declare that the research was conducted in the absence of any commercial or financial relationships that could be construed as a potential conflict of interest.

## Abbreviations

CMOS: complementary metal oxide semiconductor
CF amplitude: central footprint peak amplitude
CF area: central footprint area
CF location: central footprint location
DIV: day *in vitro*
HD-MEA: high-density microelectrode array
PCA: principal-component analysis.
